# A Comparative Study of Microbial Fuel Cells and Microbial Electrolysis Cells for Bioenergy Production from Palm Oil Mill Effluent^§^

**DOI:** 10.17113/ftb.63.02.25.9020

**Published:** 2025-06

**Authors:** Abu Danish Aiman Bin Abu Sofian, Vincent Lee, Henry Marn Jhun Leong, Yeong Shenq Lee, Guan-Ting Pan, Yi Jing Chan

**Affiliations:** 1 Department of Chemical and Environmental Engineering, University of Nottingham Malaysia, Broga Road, 43500, Semenyih, Selangor, Malaysia; 2 Department of Chemical Engineering, University of Manchester, Manchester, M13 9PL, United Kingdom; 3 College of Science, Health, Engineering & Education, Murdoch University, 90 South Street, Murdoch, WA 6150, Australia

**Keywords:** microbial electrolysis cells, microbial fuel cells, hydrogen, palm oil mill effluent, proton exchange membrane, bioenergy

## Abstract

**Research background:**

The increasing environmental concerns due to fossil fuel consumption and industrial wastewater pollution necessitate sustainable solutions for bioenergy production and wastewater treatment. Palm oil mill effluent (POME), a high-strength industrial wastewater, poses significant environmental challenges. Microbial electrolysis cells (MEC) and microbial fuel cells (MFC) offer promising avenues for bioenergy recovery from such wastewaters.

**Experimental approach:**

Dual-chamber H-type reactors equipped with proton exchange membranes were used to separately evaluate the performance of MEC and MFC in the production of bioenergy from POME. Hydrogen production and chemical oxygen demand (COD) removal in MECs were evaluated at different applied voltages and influent COD expressed as oxygen concentrations, while in MFCs the effect of external resistance on power output and COD reduction was investigated. Response surface methodology (RSM) was used to optimise these operational parameters for maximum bioenergy recovery and efficient wastewater treatment.

**Results and conclusions:**

The results showed that the efficiency of hydrogen production and COD removal in MECs were maximised at low influent COD value and low voltage supply. The MEC effectively produced hydrogen and treated industrial wastewater, while the MFC successfully produced electricity and reduced COD. Field emission scanning electron microscopy confirmed the formation of biofilms on the electrodes, indicating active microbial communities involved in the production of bioenergy. A trade-off between power density and COD removal efficiency in MFCs was observed, with medium resistance values yielding maximum power output. The integration of MEC and MFC showed potential for treating high-strength industrial wastewater like POME, offering a greener and more energy-efficient approach.

**Novelty and scientific contribution:**

This study demonstrates the potential feasibility of integrating MEC and MFC technologies for simultaneous bioenergy production and wastewater treatment from POME. It extends the knowledge in biochemical engineering by optimising operational conditions for improved bioenergy recovery and highlights the role of microbial communities in bioelectrochemical systems. The results form a basis for future research on sustainable bioenergy production and contribute to efforts towards environmental sustainability.

## INTRODUCTION

Malaysia is among the world’s main palm oil exporters (*1*, *2*). However, the production of palm oil leads to a huge amount of wastewater, namely palm oil mill effluent (POME) (*3*). For every tonne of palm oil produced, approx. 5.0 to 7.5 tonnes of POME is produced (*4*). POME is a highly polluting wastewater that contains a high amount of organic matter, where the chemical oxygen demand (COD) values range from 45 000 to 65 000 mg/L, the biochemical oxygen demand (BOD) values from 18 000 to 48 000 mg/L and the concentration of oil and grease is more than 2000 mg/L (*5*). Its composition includes a range of readily fermentable carbohydrates and volatile fatty acids such as glucose, fructose, xylose, arabinose, acetate and butyrate, which are favourable substrates for fermentative and electrogenic bacteria (*6*).

Anaerobic digestion is the most common way to treat POME due to its higher treatment effectiveness and high energy recovery (*7*-*9*). However, conventional anaerobic digestion faces several limitations in fully recovering the energy potential of POME. These include low and inconsistent methane yields due to the accumulation of inhibitory by-products, long hydraulic retention times, and sensitivity to fluctuations in POME composition, which can reduce process stability and energy recovery efficiency. To overcome these limitations, integrating bioelectrochemical systems such as microbial electrolysis cells (MEC) and microbial fuel cells (MFC) has gained attention. However, given the distinct electrode architectures and electron transfer pathways of MEC and MFC (*10*-*12*), a dedicated focus on the performance of each system in POME valorisation is necessary to fully exploit their respective potentials. These systems increase energy conversion by utilising electroactive microbes to generate hydrogen (in MEC) or electricity (in MFC) directly from organic matter. MEC can recover more energy by driving hydrogen production with minimal external energy input, while MFC enable simultaneous wastewater treatment and production of electricity. Moreover, both systems support additional resource recovery, including nutrients and biosolids, making them attractive for sustainable POME management. A comparison of MEC with MFC under identical operating conditions will clarify the trade-offs between hydrogen and electricity yields, COD removal efficiency, and operational costs, thereby guiding the choice of the optimal technology for large-scale POME treatment. With over 400 palm oil mills operating in Malaysia, the promotion of such integrated systems is critical to addressing the environmental and energy problems caused by the industry (*13*).

The primary objective of this study is to evaluate the feasibility and potential of an integrated MEC-MFC system for the treatment of POME. The novelty of this research is in the comparative analysis of individual dual-chamber H-type MEC and MFC for the production of hydrogen and electricity, respectively, while treating high-strength wastewater, with suggestions for future integration. The investigation focuses on the influence of applied voltage and influent COD values on hydrogen production and COD removal efficiency within the MEC, as well as the effect of resistance on MFC power density and COD removal. Response surface methodology (RSM) is used to optimise the MEC system by analysing the interactions between applied voltage and COD value. Field emission scanning electron microscopy (FE-SEM) was used to confirm microbial presence within MEC and MFC systems, providing evidence of electroactive microbial activity. By addressing challenges related to internal resistance and effective POME treatment, this study aims to contribute to the development of sustainable bioenergy technologies and promote resource recovery in the palm oil industry.

## MATERIALS AND METHODS

### Wastewater collection

Palm oil mill effluent (POME) and sludge samples were collected from a palm oil mill in Pahang, Malaysia, and stored under anaerobic conditions at a refrigeration temperature of 4 °C before use. The collected POME had a chemical oxygen demand (COD) expressed as oxygen concentration of 72 200 mg/L, while the sludge had a COD of 20 200 mg/L.

### Reactor set-up

A dual-chamber H-type reactor (Wente Experimental Ware, Changshu, PR China) was used to operate both MEC and MFC. The reactor consisted of two identical reagent bottles, each with a plastic cap and a volume of 300 mL. The top of the reactor was made of rubber so that tubes and wires can be inserted while the reactor remains airtight. Each chamber has three main ports for circulation or extraction purposes.

Both anode and cathode chambers were separated by a Nafion membrane, which is a proton exchange membrane (PEM) (Nafion 117; Dupont, Wilmington, DE, USA) where only free protons (H^+^) can pass through (*14*). The Nafion membrane used had dimensions of 49 mm×49 mm, resulting in a working area of 24.01 cm^2^. The Nafion membrane was pre-treated by immersion in *w*(H_2_O_2_)=5 % at 80 °C for 1 hour, followed by deionised water for 30 min, then in *w*(H_2_SO_4_)=5 % at 80 °C for 1 hour, and finally rinsed again in deionised water for 30 min before use (*15*).

Graphite plate electrodes with dimensions of 70 mm (length) × 27 mm (width) × 2 mm (thickness) were used in both the anode and cathode chambers in MEC and MFC systems. Graphite was chosen for its excellent performance in open circuit voltage, current density and power generation in microbial electrochemical systems (*16*). The total surface area of each rectangular electrode was 38.88 cm^2^ after accounting for the glued wire area, the effective area was approx. 35 cm^2^, which was used in all calculations of the performance. The electrode spacing was maintained at approx. 8 cm to reduce internal resistance, consistent with the setup reported by Almatouq and Babatunde (*17*). The solution in the anode chamber was circulated using a magnetic stir bar, while the cathode chamber solution was circulated using a peristaltic pump (BT100J-1A; Longer Pump, Baoding, PR China). For MEC operation, a 1000 Ω resistor was connected between the cathode and the negative terminal of the DC power supply.

### Inoculation stage

Biofilms were cultivated on the anodes of both MEC and MFC during the inoculation process, with some procedures common to both systems. Prior to inoculation, the pH of all solutions in both anode and cathode chambers was adjusted to neutral (pH=7) using either sodium hydroxide or hydrochloric acid. In order to create anaerobic conditions in the anode chambers, pure nitrogen gas was purged for at least 20 min. In addition, all anolytes were supplemented with growth nutrients per litre of chamber volume: 0.984 g sodium acetate, 0.039 g potassium chloride, 0.15 g glucose, and 10 mL of tap water as a source of trace minerals. The inoculation process in both systems continued for a minimum of one month to allow the formation of stable and mature biofilms, which are essential for effective electron transfer and system performance.

In the MEC, the anolyte consisted of POME diluted with deionised water at a volume ratio of 1:50, and a 0.05 M phosphate buffer in a volume ratio of 1:1 (*17*). The phosphate buffer used in the MEC anolyte was prepared with phosphate-buffered saline (PBS) in the following concentrations: 5.79 mM NH_4_Cl, 17.77 mM NaH_2_PO_4_·H_2_O, 32.23 mM Na_2_HPO_4_ and 1.74 mM KCl. To initiate biofilm formation, an external voltage of 0.9 V was applied to the system (*18*). The voltage was monitored continuously, and a drop towards 50 mV was used as an indicator of sufficient biofilm development, based on preliminary observations and supported by results from Wang *et al.* (*19*), who found that voltage responses in MEC correlate with changes in biofilm conductivity and activity. They reported that biofilm conductivity increases with applied voltage and deviates from Ohm’s law above 100 mV, indicating shifts in electron transfer mechanisms during biofilm growth. This suggests that sustained low voltages reflect electroactive and mature biofilms. Once this condition was reached, half of the anolyte was replaced with fresh phosphate buffer and substrate to replenish nutrients and maintain stable performance. Simultaneously, the external resistor was adjusted to 10 Ω to maintain higher current flow and support continued biofilm activity.

In the MFC, the anolyte consisted of a mixture of 70 % diluted POME and 30 % sludge, also in a 1:1 volume ratio with the phosphate buffer. The phosphate buffer used in the MFC anolyte had the same composition as described for the MEC. The anodes were connected to the positive pole of the power source, while the cathodes were connected to the negative pole *via* a 1 kΩ resistor.

### Experimental procedures

Both MEC and MFC experiments were conducted in batch mode for three consecutive operating days and repeated twice weekly. Initial measurements of COD, pH and conductivity were taken from both chambers at the start of each run and final measurements were recorded after the batch cycle.

The anode chamber solution was prepared by mixing 200 mL of diluted POME with 200 mL of 0.05 M phosphate buffer, resulting in a total volume of 400 mL. COD concentration was adjusted by varying the ratio of raw POME to deionised water in the dilution step. The use of diluted POME provided a natural source of microorganisms, including bacteria, essential for the bioelectrochemical processes (*20*, *21*). Despite the absence of added sludge, the organic matter and microbial load of POME supported effective microbial activity. This setup was crucial for achieving the desired electrochemical reactions and demonstrated the practical application of using waste effluents directly in MFC/MEC systems. A volume of 300 mL of the prepared solution was used as the anode chamber solution, while the remaining 100 mL was used for various tests.

#### MEC operation

**Fig. S1:**
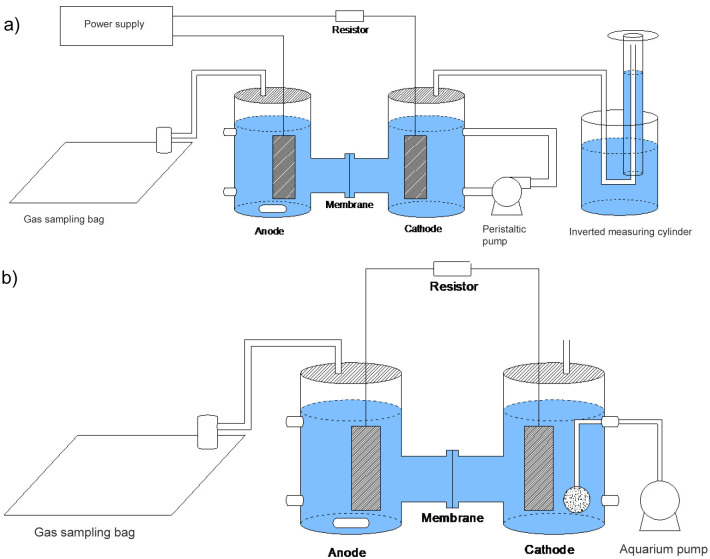
Schematic diagram of: a) microbial electrolysis cells (MEC) and b) microbial fuel cells (MFC)

The preliminary experiment was conducted at different applied voltages (0.2, 0.7 and 1.2 V). Additionally, different COD values (200, 1000 and 2000 mg/L) of the anode chamber solution were used. The schematic diagram of the MEC reactor is shown in Fig. S1a. The hydrogen gas produced in the cathode chamber was collected in an inverted 100-mL measuring cylinder submerged in water and the volume was measured. Any gas generated in the anode chamber was collected in a gas sampling bag. The resistor connecting the cathode electrode to the power supply was changed to 10 Ω for this experiment. The hydrogen gas collected in the inverted measuring cylinder was transferred into a gas sampling bag after each experiment. At the end of each batch cycle, the reactors were exposed to air to inhibit the formation of methanogens and reduce the production of methane gas (*22*). This exposure was carefully controlled to minimise damage to the electroactive populations.

#### MFC operation

The experimental setup of the MFC is shown in Fig. S1b. The configuration of the MFC differed from that of the MEC, as the cathode chamber was aerated. The experiment was conducted using different resistor resistances (1, 3.3, 5.1, 10 and 41 kΩ) to evaluate the performance of the MFC under different external loads. These resistances were selected to represent a range of typical loads used in MFC experiments, allowing the analysis of how different resistances affect COD removal efficiency and voltage. This approach is consistent with established methods in MFC studies. For instance, Potrykus *et al.* (*23*) evaluated resistances from 0.12 to 3.3 kΩ and Li and Chen (*24*) evaluated 0.01 to 10 kΩ. Kamau *et al.* (*25*) evaluated a wider range from 0.001 to 33 kΩ and observed that the generated power increased from 0.00002 to 0.003131 mW when the external resistance was varied from 1 Ω to 33 kΩ on day 6. Evaluating a wide range of resistances is crucial to a comprehensive understanding of MFC performance characteristics. It helps to determine the optimal resistance for maximum power density to ensure efficient operation of the system. Additionally, it provides insights into the operational limitations and potential areas for improvement in MFC design and operation. Therefore, a wide range of 1 to 41 kΩ was evaluated in this study. The gas produced in the anode chamber was collected in a gas sampling bag *via* a hose connected through the top of the reactor. In this experimental setup, the cathode chamber was left open to allow the escape of air.

### Analytical methods and calculations

Hydrogen produced in the MEC was collected in an inverted measuring cylinder immersed in water *via* a tube. The volume of produced hydrogen was determined by looking at the water level in the measuring cylinder and the results were recorded every day. The produced hydrogen gas was kept in a gas sampling bag after the experiment. Gas chromatography (GC) (Clarus 580; PerkinElmer, Waltham, MA, USA) was used to analyse the gas composition to confirm that hydrogen gas was produced.

The voltage across the resistor in both MEC and MFC was recorded every day using a digital multimeter. The current across the reactor was calculated using Ohm’s law based on the voltage across the resistor, where *I=V/R*. However, in MEC, the resistor causes an additional voltage loss in the system. Hence, the actual voltage, *E*_ap_, applied to the reactor was smaller than the voltage supplied by the power source, *E*_ps_ (*22*). The actual applied voltage can be determined using the following equation:


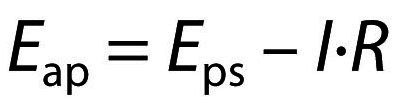
 /1/

where *I* is the current in amperes across the resistor and *R* is the resistance of the resistor.

Current density and power density were used to determine the performance of the MFC. The current density (*j*) was calculated using the following formula:


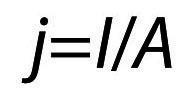
 /2/

where *I* is the current in amperes and *A* is the surface area of the anode in m^2^. The power density was calculated using the following formula:


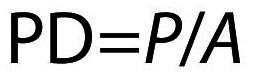
 /3/

where *P* is the voltage (*V*) × current (*I*) and *A* is the surface area of the anode electrode in m^2^.

The COD values of the anode solution in both MEC and MFC were measured before and after every experimental run to determine the COD removal efficiency (*η*_COD_) using the following equation:


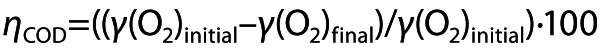
 /4/

A spectrophotometer (DR2800: Hach, Loveland, CO, USA) was used to measure the COD values of the solution in the Hach high range COD digestion vials at a range of 20-1500 mg/L. The conductivity of the anode and cathode solutions in both MEC and MFC was measured using a conductivity meter (CON2700; Eutech, Singapore) before and after every experimental run.

Coulombic efficiency (CE) was calculated using the following equation:


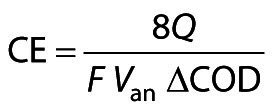
 /5/

where *Q* is the number of Coulombs, *F* is the Faraday constant, *V*_an_ is the volume of the reactor in L, and ΔCOD is the amount of COD removed (*26*).

### Statistical analysis

In the MEC experiment, RSM was used to understand the effect of different parameters on the response of the system and optimise its performance (*17*). Design-Expert software v. 13.0 (*27*) was used to design, analyse and optimise this experiment. Two factors, namely COD value (A) and applied voltage (B), were considered, while the responses were hydrogen production rate and COD removal efficiency. Analysis of variance (ANOVA) was carried out to evaluate the regression of the fitted model and the R^2^ value was used to determine the goodness-of-fit of the model. The significance of the model was evaluated based on the model F-value and the significance of model terms was determined using the p-value at a confidence level of 95 %. The software was used to identify the optimised operating conditions for the MEC system. To examine the effects of COD concentration and applied voltage on the hydrogen production rate and COD removal efficiency, a central composite design (CCD) was used. CCD is chosen in this study as it is a widely used experimental design method that analyses both linear and nonlinear relationships between variables (*11*). This approach allows a systematic investigation of the relationship between the experimental factors and the desired responses and helps to identify optimal conditions for improved bioenergy production and wastewater treatment efficiency in the MEC system.

## RESULTS AND DISCUSSION

### Microbial electrolysis cells

The preliminary results in Table 1 show that the maximum cumulative hydrogen production observed in this experiment was 104.4 mL and corresponds to a hydrogen yield of 3.135 m^3^ of H_2_ per kg of COD_removed_. This value is higher than those reported by Marone *et al.* (*28*) (1.609 m^3^/kg) and Khongkliang *et al.* (*29*) (0.236 m^3^/kg). The highest hydrogen production was observed at lower initial chemical oxygen demand (COD) values (≤315 mg/L) and moderate applied voltages (0.199–0.652 V), while higher COD values (790–1930 mg/L) and higher voltages (0.757–1.192 V) led to reduced yields. These results suggest that the hydrogen production rate is affected by both the initial COD value and the applied voltage.

**Table 1:** Preliminary results obtained in microbial electrolysis cells (MEC) at different experimental conditions

**Parameter**			***N*(MEC batch)**									
			1	2	3	4	5	6	7	8	9	10
**COD as *γ*(O_2_)/(mg/L)**	Initial		150	206	315	770	790	915	980	1635	1855	1930
	Final		138	95	185	635	625	1085	815	1560	1905	1920
***η*_COD_/%**			8	53.88	41.27	17.53	20.89	N/A	16.84	4.59	N/A	0.52
**COD_removed_** ***γ*(O_2_)_initial_-*γ*(O_2_)_final_**			12	111	130	135	165	N/A	165	75	N/A	10
**_supplied_/V**			1.198	0.679	0.199	0.176	1.343	1.104	1.198	0.758	0.661	1.187
**_resistor_/mV**			5.75	1	0.1	0.2	2.25	22.5	5.75	1	9.5	2
**_applied_/V**			1.192	0.678	0.199	0.176	1.341	1.081	1.192	0.757	0.652	1.185
***I*/mA**			0.575	0.1	0.01	0.02	0.255	2.25	0.575	0.1	0.95	0.2
***j*/(mA/m^2^)**			152.12	26.46	2.65	5.29	59.52	595.24	152.12	26.46	251.32	52.91
***V*(cumulative H_2_ production)/mL**			37.8	0.6	104.4	92.4	17.4	69.6	37.8	33	93	37.8
***Q*(H_2_ production)/(mL/day)**			12.6	0.2	34.8	30.8	5.8	23.2	12.6	11	31	12.6
***V*(H_2_ production)/*m*(COD_removed_)/(m^3^/kg)**			N/A	0.167	3.135	2.369	0.43	1.406	N/A	0.667	4.133	N/A
**Coulombic efficiency/%**			N/A	59.7	0.65	1.1	11.94	97.69	N/A	4.34	0.91	N/A
**pH**		Anode chamber_final_	6.74	6.92	6.68	6.44	6.2	6.76	6.77	6.14	6.29	6.26
		Cathode chamber_final_	6.98	6.98	7.01	7.02	7.01	7.27	7.32	7.01	7.18	7.15
		pH_cathode chamber final_-pH_anode chamber final_	0.24	0.06	0.33	0.58	0.81	0.51	0.55	0.87	0.89	0.89

COD=chemical oxygen demand, N/A=not applicable

Lower COD values can mitigate substrate inhibition and promote more effective microbial activity and bioelectrochemical processes, as high COD values can be detrimental to microorganisms (*30*). Additionally, using lower applied voltages can reduce competitive reduction reactions at the cathode, thus improving hydrogen production efficiency. High applied voltages can induce oxidative stress, which may damage cells and diminish microbial growth and activity. This stress can restrict substrate oxidation, decrease proton (H^+^) availability and ultimately reduce overall hydrogen production (*31*).

These results also highlight the importance of optimising operating parameters to achieve a balance between high COD removal efficiency, hydrogen production rate and cost-effectiveness. While a voltage of 0.2 V resulted in significant hydrogen production in this study, this may be due to specific experimental conditions and might not apply to all microbial electrolysis cell (MEC) systems. According to Tan *et al.* (*32*), the typical range of external voltages for MECs is 0.2 to 0.8 V to balance overpotential and internal resistance, which can affect hydrogen production efficiency. Logan *et al.* (*22*) further support this by noting that higher applied voltages increase the electrical energy input per amount of hydrogen produced (kWh/m^3^) and emphasise the need to minimise energy loss while maintaining production rates. Additionally, Logan *et al.* (*22*) indicated that microbial electrolysis reactions generally start at applied voltages above 0.2 V, corresponding to an energy requirement of 0.43 kWh/m^3^ (at 100 % cathodic hydrogen recovery). Therefore, while these findings support the potential for lower voltage operation, further studies are necessary to generalise the results and optimise operating parameters for different MEC configurations to ensure cost-effective hydrogen production. This includes minimising electrical energy input to reduce overall operational costs and increase the economic viability of MEC technology.

The highest Coulombic efficiency (CE) achieved in this experiment was 97.69 %, which occurred at a COD value of 790 mg/L and a voltage of 1.104V. Some CE values were unavailable due to the unchanged COD value after the experiment. The CE values ranged from 0.91 to 97.69 %, confirming the results of Hari *et al.* (*33*), where CE ranges from 50 to 90 %, depending on operational conditions and substrate type. It was observed that CE was affected by the current across the reactor and the amount of COD removed. Specifically, CE increased as the current across the reactor increased, while higher COD removal resulted in a decrease in CE. The microbial community can efficiently utilise the available substrate at lower COD values, leading to higher CE. In contrast, at higher COD values, substrate inhibition may reduce microbial efficiency and reduce CE. Similarly, higher voltages can improve microbial electroactivity, but they may also increase competition for electrons and trigger side reactions, leading to lower CE. These factors contribute to the observed variability in CE and highlight the complexity of microbial electrochemical processes and the need for careful optimisation of operational parameters to achieve consistently high CE in MEC systems.

Furthermore, a direct correlation was observed between the COD value in the anode chamber and the pH difference between the anode and cathode chambers (Table 1). Higher COD values resulted in greater pH disparities between the two chambers, leading to increased potential loss (*17*). To address this issue, a phosphate buffer solution was used in the experiment to maintain a balanced pH within the system. For each 300-mL chamber, approx. 50-100 mL of phosphate buffer was used based on preliminary tests to stabilise the pH. However, it is important to note that the use of phosphate buffer solution in large-scale MEC applications poses a significant challenge due to its high cost and the potential environmental impact associated with the disposal of large volumes of used phosphate buffer solution (*34*). These considerations necessitate further investigation and development of alternative pH control strategies for MEC scaling-up efforts.

To further investigate the effects of the independent variables (COD concentration and applied voltage) on hydrogen production rate and COD removal efficiency, nine experimental conditions based on the central composite design (CCD) were carried out in the laboratory. Based on the preliminary results, the ranges of influent COD value (mg/L) and voltage were determined (Table 1). According to the CCD, the observed experimental results regarding hydrogen production in the MEC are shown in Table 2.

**Table 2:** Experimental conditions and results for central composite design (CCD)

**Run**	**Parameter**		**Response**		
	A: Influent CODas *γ*(O_2_)/(mg/L)	B: *V*_supplied_/V	*V*(H_2_ cumulative production)/mL	*Q*(H_2_ production)/(mL/day)	*η*_COD_/%
**1**	980	0.758	33.0	11.0	16.84
**2**	1930	0.170	67.8	22.6	0.52
**3**	315	0.176	92.4	30.8	41.27
**4**	206	0.199	104.4	34.8	53.88
**5**	770	1.343	17.4	5.8	17.53
**6**	980	0.758	33.0	11.0	16.84
**7**	315	0.176	92.4	30.8	41.27
**8**	206	0.199	104.4	34.8	53.88
**9**	770	1.343	17.4	5.8	17.53

COD=chemical oxygen demand

### MEC cumulative hydrogen production

The ANOVA results (Table S1) showed a significant two-factor interaction (2FI) model, suggesting that both, influent COD expressed as oxygen concentration (A) and voltage supply (B), as well as their interaction (AB), affected hydrogen production rate. The high R^2^ value of 0.9918 and adjusted R^2^ value of 0.9868 indicate that the model is well-fitted to the data, with a strong degree of fitness. This suggests that the model can explain a significant portion of the variability in the hydrogen production rate.

**Fig. 1:**
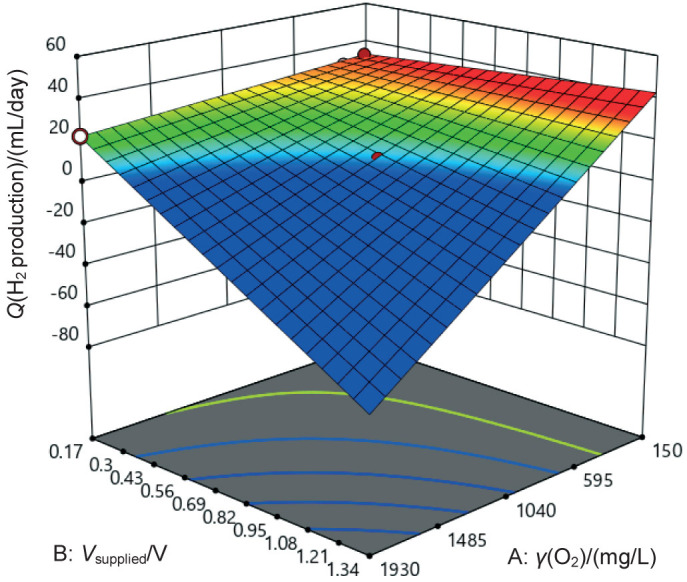
Response surface of hydrogen production rate (*Q*) as a function of influent chemical oxygen demand (COD) value expressed as concentration of oxygen and applied voltage

The response surface plot (Fig. 1) offers a visual representation of the relationship between the two factors and cumulative hydrogen production. It illustrates the influence of the factors on the response variable and can be used to identify optimal operating conditions for maximising hydrogen production. All the model terms were statistically significant, as indicated by the low p-values (p<0.1) (Table S1). This suggests that each factor and its interactions significantly affect cumulative hydrogen production. The model F-value of 201 indicates that the model was significant, with a 0.01 % chance that a large F-value could occur due to noise. The following model explains the hydrogen production rate *Q*:


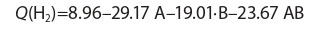
 /6/

where A is influent COD expressed as oxygen concentration (in mg/L) and B is the voltage supply (in V).

A negative term in Eq. 6 indicates an inverse relationship between the manipulated variables (influent COD value and voltage supply) and the response variable (hydrogen production rate). This shows that both higher COD values and higher voltages reduce hydrogen production. The significant interaction term between A and B (−23.67 A B) suggests a synergistic negative effect, meaning that when both A and B are present, the reduction in hydrogen production is greater than the sum of their individual effects. Therefore, optimising both parameters simultaneously to maximise hydrogen production is essential.

This result is consistent with the 3D surface graph (Fig. 1), where hydrogen production increased at low COD values and moderate voltage. This may be attributed to more favourable microbial conditions under low organic load, when excessive substrate or voltage could disrupt microbial balance or induce inhibitory effects (*24*). These findings highlight the need for careful tuning of both variables to maintain microbial performance and maximise hydrogen yield.

### COD removal efficiency of MEC

The ANOVA results for COD removal efficiency (*η*_COD_) are shown in Table S1. Similar to the hydrogen production rate, a high R^2^ value of 0.9639 and an adjusted R^2^ value of 0.9518 are obtained, which indicates a good degree of fitness for the model. This suggests that the model can accurately explain the variability in COD removal efficiency. The significance of the model is further supported by the model F-value of 80.05, which indicates a 0.01 % chance that such a large F-value could occur due to noise.


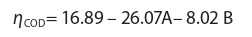
 /7/

where A is influent COD expressed as oxygen concentration (in mg/L) and B is the voltage supply (in V).

The linear model presented in Eq. 7 describes the relationship between COD removal efficiency and the factors that affect it. This model indicates that lower COD values and lower voltage improve organic degradation. This is because high COD values may exceed microbial degradation capacity, while higher voltages could negatively affect microbial viability (*22*). The negative coefficients in the equation indicate an inverse effect between the manipulated variables (influent COD value and voltage supply) and the response variable (COD removal efficiency). This suggests that as the influent COD value and voltage supply decrease, the COD removal efficiency increases.

**Fig. 2:**
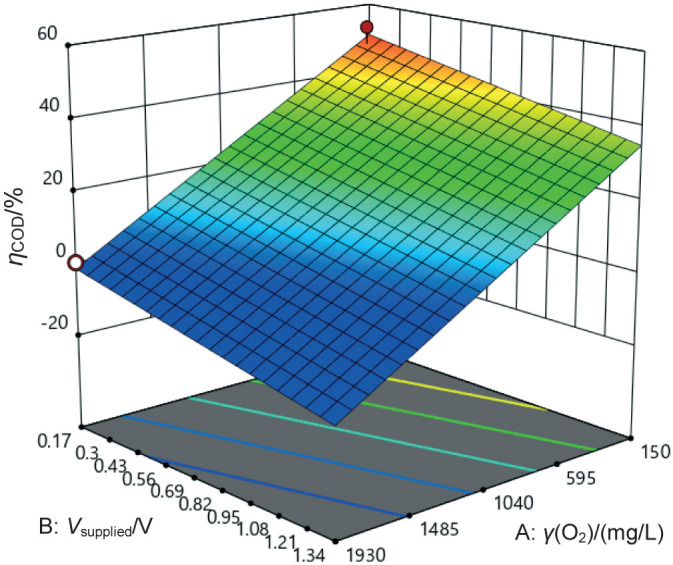
Response surface of chemical oxygen demand (COD) removal efficiency as a function of influent COD value expressed as oxygen concentration and applied voltage

This result is consistent with the observed relationship in the hydrogen production rate (Fig. 1). The 3D surface graph in Fig. 2 illustrates this relationship and shows that higher voltage supply leads to lower COD removal efficiency. This is possibly attributed to the potential negative effects of high voltage on microorganisms, which can hinder their ability to effectively degrade organic matter (*35*).

Furthermore, the COD removal efficiency decreases as the COD expressed as O_2_ concentration in the solution increases. This can be attributed to the limited time available for the microorganisms to degrade higher concentrations of organic matter, resulting in lower efficiency. Therefore, lower COD values in the solution are associated with higher COD removal efficiency. To achieve higher COD removal efficiency at higher COD values, longer retention times may be required to provide sufficient opportunity for complete degradation.

In summary, these findings highlight the critical role of controlling the influent COD values and voltage supply to achieve optimal performance in MEC. By decreasing the influent COD value and selecting suitable voltage, significant improvements can be achieved in both COD removal efficiency and hydrogen production rate. This not only improves the overall system efficiency but also reduces energy consumption. Building on these results, further optimisation strategies were implemented, which will be discussed in the following section.

### MEC parameter optimisation

The empirical model represented by Eqs. 6 and 7 was evaluated using the Design-Expert software (*27*) to determine the optimal conditions for maximising hydrogen production and COD removal efficiency. Table S2 shows the results of the optimisation process, where the initial COD values and voltage supply were adjusted within the specified operating range to achieve the maximum hydrogen production rate and COD removal efficiency. The optimal operating conditions were identified at a COD value of 150 mg/L and a voltage supply of 0.338 V, with a high desirability value of 0.966 (Table S2). The results suggest that the hydrogen production rate and COD removal efficiency can be maximised by employing low influent COD values and a low voltage supply. This is consistent with previous reports stating that hydrogen production is achievable at applied voltages as low as 0.2 V, although values below 0.3 V may lead to low hydrogen production rates and unstable system performance (*36*). Operating at lower voltages offers practical advantages, including reduced energy consumption and lower operational costs. Furthermore, minimising power input aligns with sustainability goals by reducing carbon emissions associated with MEC operation.

### Properties of the MEC biofilm on the anode

**Fig. 3:**
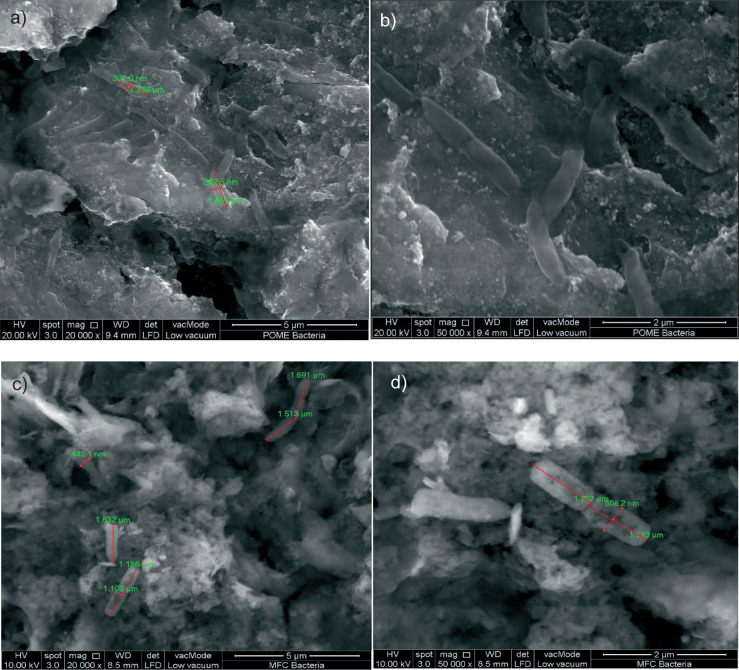
Bacteria found on the anode electrode of microbial electrolysis cells (MEC) at: a) 20 000×, b) 50 000× magnification. Bacteria found on the anode electrode of microbial fuel cells (MFC) at: c) 20 000× and d) 50 000× magnification

Field emission scanning electron microscopy (FE-SEM) was used to examine the biofilm on the anode electrode (Fig. 3) and it showed bacterial presence at a magnification of 20 000× as shown in Fig. 3a. While FE-SEM is a valuable tool for visualising biofilm structure and bacterial colonisation, it does not provide definitive species identification. For example, Mejía-López *et al.* (*37*) used FE-SEM in their MEC studies to show that bacteria preferentially attached to the particles on the electrode surface, with populations showing uniform morphology and distribution.

The observation of bacterial biofilms supports the hypothesis that microbial processes contribute to hydrogen production. Previous studies by Wang *et al.* (*38*), Liu *et al.* (*39*) and Logan *et al.* (*22*) have reported the presence of *Pseudomonas* spp. and *Shewanella* spp. in similar MEC systems. Liu *et al.* (*39*) specifically noted that rod-like bacteria are likely the functional strains with electrochemical activity. This was supported by single-strand conformation polymorphism (SSCP) analysis, which identified *Pseudomonas* sp. and *Shewanella* sp. as dominant during the hydrogen production stage. The rod-shaped bacteria observed in this study (Fig. 3a and Fig. 3b) suggest that similar species could be present. This supports the established knowledge of microbial communities in MECs, despite the lack of additional molecular identification techniques.

Acknowledging the limitations of FE-SEM, further research involving molecular methods, such as PCR or 16S rRNA sequencing, is necessary for precise species identification. However, FE-SEM effectively demonstrates microbial biofilm formation and supports the role of microbial activity in hydrogen production. This preliminary observation provides a foundation for subsequent studies aimed at characterising specific bacterial species and their contributions to the MEC process.

### Microbial fuel cells

The microbial fuel cell (MFC) experiment was conducted using different resistor resistances (Table 3). It is important to note that the composition of the anode solution in the MFC differs from that of a MEC. In the MFC, the anode solution is prepared by combining raw sludge and diluted palm oil mill effluent (POME). To ensure consistency, the quantities of raw sludge, raw POME and deionised water were kept constant throughout the experiment. The focus of this experiment was solely on manipulating the resistor by using different resistances. By maintaining consistency in the anode solution and varying the resistor resistances, the aim of the study was to explore the effect of this parameter on the performance of the MFC.

**Table 3:** Results obtained from microbial fuel cells (MFC)

**Parameter**		**MFC batch**				
		1	2	3	4	5
***V*_resistor_/mV**		14	60	195	291	338
***V*(*t*=4 day)/mV**		10	117	201	268	327
***I*/mA**		0.0100	0.0355	0.0394	0.0268	0.0076
***P*/µW**		0.1000	4.1482	7.9218	7.1824	2.4867
***j*/(mA/m^2^)**		2.65	9.38	10.43	7.09	2.01
**(*P*/*A*)/(µW/m^2^)**		26.46	1097.40	2095.70	1900.11	657.86
***R*_resistor_/Ω**		1000	3300	5100	10000	43000
**Coulombic efficiency/%**		0.036	0.231	0.076	0.074	N/A
***η*_COD_/%**		22.47	31.58	62.71	68.42	64.85
**pH**	Anode chamber _final_	7.13	7.31	7.29	7.39	7.41
	Cathode chamber_final_	7.41	7.51	7.51	7.51	7.47
	pH_cathode chamber final_-pH_anode chamber final_	0.28	0.20	0.22	0.12	0.06

### MFC power and current density profiles

The power density of a system is often used to compare its power output. The power output of a system is determined by the projected area of the anode, where biological reactions occur (*40*). The maximum power density recorded was 2095.70 mW/m^2^ at a current density of 10.43 mA/m^2^ and a resistance of 5100 Ω (Batch 3, Table 3). Power density is observed to increase as the current density in the MFC increases. While relatively low, this value is comparable to those reported by Chonde (*41*), which is 2.87 kW/m^3^, and is slightly lower than the values reported by Zain *et al.* (*42*), who achieved a maximum power density of 9053 mW/cm^2^ using activated sludge. Similarly, Fischer *et al.* (*43*) achieved 16.2 mW/m^2^.

Previous MFC studies reviewed by Tan *et al.* (*32*) and Obileke *et al.* (*44*) achieved higher power densities, ranging from 78 to 460 mW/m^2^. These higher values can be attributed to differences in electrode materials, operational conditions (temperature and pH), pure culture or mixed culture inoculum, proton exchange system (PES) and design and configuration of MFC reactors (*44*). Daud *et al.* (*45*), for example, produced a novel porous clay earthenware (NCE) as a low-cost separator to replace the high-cost proton exchange membrane (PEM) (Nafion 117). The highest power and current densities recorded in their experiments were (2250±21) mW/m^2^ and 6.0 A/m^2^, respectively, using the NCE low-cost separator and 30 % starch powder under batch mode operation.

The lower power densities obtained in this study are primarily due to non-optimised conditions and less conductive electrode materials. The methodology focused on exploring the basic principles of MFC operation and integration with MEC rather than optimising power output. Future research will aim to improve the performance by optimising conditions and exploring advanced materials and reactor designs.

**Fig. 4:**
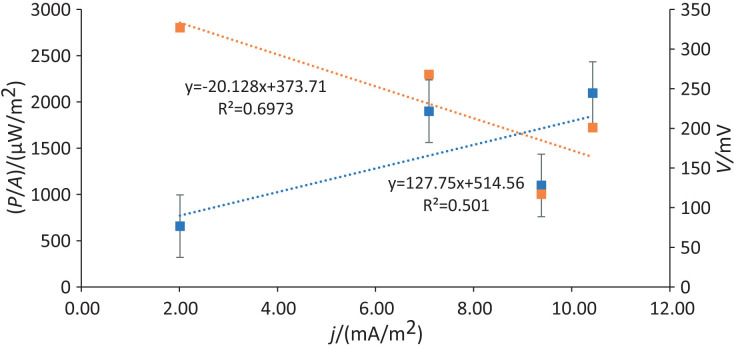
Polarisation (orange) and power density (blue) curves

The polarisation curve, also known as the voltage-current curve (Fig. 4), is essential for understanding the operation of MFC, as it represents the voltage at different current densities (*40*). This curve is divided into three distinct regions: activation loss, ohmic loss and concentration loss (*22*). The first region, characterised by a steep decrease in voltage at zero current, reflects activation loss. This loss is due to the high energy barrier needed to initiate oxidation or reduction reactions, where electrons are transferred from bacteria to the anode surface (*22*). Strategies such as increasing the anode surface area and using materials with higher catalytic activity can help to minimise activation loss and improve electron transfer efficiency.

The second region of the polarisation curve corresponds to ohmic loss, where the voltage drop becomes more constant and shows a linear relationship with the current. This loss results from the resistance of the solution and the membrane. In this region, the slope of the polarisation curve indicates the internal resistance of the MFC, with a lower slope being more desirable as it signifies reduced resistance and higher efficiency. Ohmic loss can be mitigated by minimising the distance between electrodes and selecting membranes with lower resistivity and higher ionic conductivity (*22*).

The final region of the curve highlights concentration loss, which occurs at higher currents and is indicated by a sharp voltage drop. This loss results from mass transfer limitations, where the rate of reaction is insufficient to maintain the flux of reactants to the electrode or the flux of products away from the electrode (*22*). To address concentration loss, potential strategies include optimising electrode design for improved mass transfer, adjusting flow rates within the MFC and using catalysts to accelerate reaction kinetics (*46*).

The internal resistance of the MFC, approx. 5.3 kΩ, was determined from the slope of the potential-current curve (Fig. 4). This internal resistance can be reduced by minimising the electrode spacing and selecting a membrane with low resistivity. The power density curve, represented by the power-current curve in Fig. 4, provides further insights into MFC performance. The power-current curve shows that power density increases with increasing current density. This trend suggests that the system power output increases as current density increases, likely due to more efficient electron transfer and energy conversion at higher currents. Understanding and analysing these curves enables the development of targeted strategies to improve MFC efficiency, ultimately improving performance in practical applications.

**Fig. S2:**
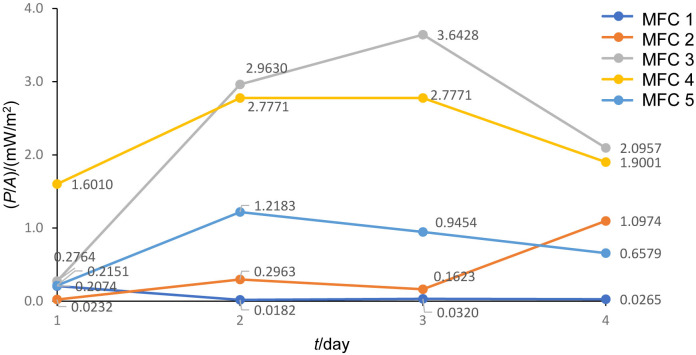
Daily power production of microbial fuel cells (MFC)

Fig. S2 shows how the power output changes over time during the operation of the MFC. It initiates with a very low power generation due to the initiation of oxidation or reduction reactions. It peaks on the second and third day of operation and gradually decreases towards the final day. This decline in power generation can be attributed to the limited reaction rate. The Coulombic efficiency of the MFC ranges from 0.036 to 0.076 %, with higher efficiencies achieved at increased current across the reactor.

### COD removal by MFC vs. applied voltage

**Fig. 5:**
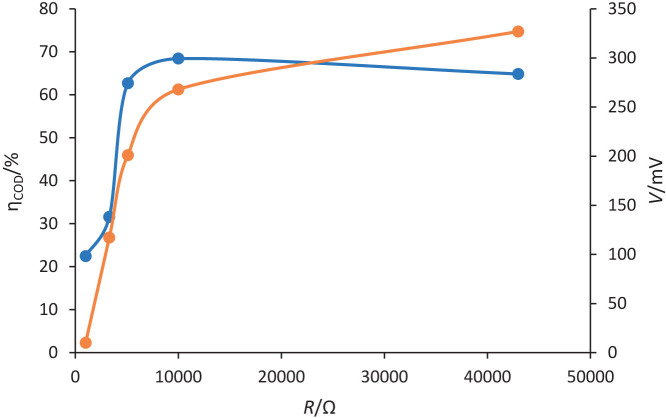
Chemical oxygen demand (COD) removal efficiency (blue) and voltage (orange) produced against resistance

Fig. 5 shows a clear relationship between resistance and voltage in the MFC system. As resistance increases, voltage also increases. This finding aligns with the proportionality between resistance and voltage described by the equation *V*=*I*∙*R*. Similarly, the COD removal efficiency shows an increasing trend up to a peak value at around 10 000 Ω, after which it decreases. This finding aligns with the results reported by Kloch and Toczyłowska-Mamińska (*47*), which showed a substantial increase of around 30 % in COD removal efficiency when the external resistance was increased from 100 to 1000 Ω. These findings highlight the critical role of external resistance as a key parameter in MFC systems. The observed increase in COD removal efficiency with higher resistance suggests that increased resistance values can stimulate improved performance in terms of organic matter degradation. However, it is important to note that beyond a certain resistance threshold, the efficiency begins to decline. This indicates the existence of an optimal resistance range that balances microbial activity and bioelectrochemical processes involved in COD removal. Therefore, the selection of the external resistance is crucial for the long-term operation of MFCs, as it directly affects the microbial community responsible for the production of bioelectricity (*48*).

This study also found that medium resistance values (5100 Ω) resulted in maximum power density, while high resistance values (10 000 Ω) led to high COD removal efficiency. This suggests a trade-off between power density and COD removal efficiency in the MFC system. This trade-off is determined by the dynamics of substrate availability, electron flow and microbial activity. At higher resistance values, the slower electron flow allows more complete oxidation of organic matter, which improves COD removal but reduces power density. Conversely, at lower resistance values, faster electron flow favours higher power density but can result in incomplete COD removal. Optimal performance in terms of power density and COD removal efficiency may require finding a balance between resistance values that maximise power output and those that improve organic matter degradation. Approaches such as dynamic resistance adjustment, optimising microbial consortia and improving electrode materials could help to balance these parameters. Further investigation is needed to explore this relationship and identify the optimal resistance range that balances power density and COD removal efficiency.

### pH dynamics of MFC

The results in Table 3 show that there was a noticeable increase in the final cathode half-cell pH compared to the initial pH value of 7. The final pH of the cathode half-cell ranged from 7.41 to 7.51. This observed increase in pH in the cathode chamber can be attributed to the cathode reaction, during which protons are consumed and hydroxyl anions are released into the solution (*26*). The final pH values for the anode half-cell ranged from 7.13 to 7.41. These changes in pH provide valuable insights into the electrochemical processes taking place within the MFC system. Maintaining proper control and regulation of pH levels in the cathode chamber is crucial for optimising the performance of the MFC. Fluctuations in pH can have significant implications for the overall efficiency and stability of the system. Further investigation and optimisation strategies can be explored to mitigate any potential negative effects associated with pH changes and maximise the performance of the MFC.

### MFC anode biofilm characterisation

FE-SEM was carried out to examine the morphology and distribution of microorganisms on the anode in the MFC, aiming to draw comparisons to those observed in the MEC. The FE-SEM provides detailed surface images that allow the visualisation of microbial structures at the nanoscale (*49*). This analysis showed the presence of rod-shaped bacteria on the anode surfaces in both MFC (Fig. 3c and Fig. 3d) and MEC (Fig. 3a and Fig. 3b) systems, consistent with observations reported by Liu *et al.* (*39*). This finding aligns with previous studies indicating that *Pseudomonas aeruginosa* can generate electricity in MFCs through the production of organic compounds such as phenazine pyocyanin, which facilitate electron transfer from the bacterium to the anode (*50*). Based on Fig. 3c and Fig. 3d, the presence of rod-shaped bacteria suggests that *Pseudomonas aeruginosa* may be among the microorganisms on the surface of MFC electrode. Although FE-SEM alone does not definitively identify bacterial species, these results provide valuable insights into the potential role of similar microbial processes in both MFCs and MECs. This assumption presents opportunities for future investigations into the utilisation of *Pseudomonas aeruginosa* and related *Pseudomonas* species in MFCs to improve bioelectrochemical energy generation. Molecular methods such as 16S rRNA sequencing should be used in future studies to confirm the bacterial species and evaluate the potential benefits and challenges of harnessing these bacteria in both MFC and MEC systems. Additionally, further research is needed to evaluate the specific mechanisms and potential benefits and challenges associated with these bacteria in bioelectrochemical systems.

### Future perspectives: Integration of MFC and MEC

Although the integration of electricity generated by MFC to power the MECs was not implemented in this study, it is proposed as a future research direction. The MEC experiment showed that a voltage of 0.3 V led to the highest hydrogen gas production and COD removal efficiency. By utilising an MFC with a 5100 Ω resistor, this voltage could potentially be provided to power the MEC, eliminating the need for an external voltage supply and reducing costs. Exploring the integration of electricity generated by MFC to power MECs for the production of hydrogen gas may offer improved results, considering the higher profit potential of hydrogen gas as bioenergy source than generating electricity. Such integration of MEC and MFC technologies could present a greener and more environmentally friendly approach to power generation and wastewater treatment. When compared to other integrated systems, such as those that combine anaerobic digestion with microbial fuel cells, MEC-MFC integration uniquely combines the production of electricity with the production of hydrogen, although similar systems also encounter challenges related to operational management and optimisation.

Future perspectives on the integration of MEC and MFC technologies involve several key considerations. Potential configurations include directly coupling MFC to power MEC, using MFC-generated electricity to drive MEC processes, and using reactor effluent from one system as influent to the other to optimise performance. Practical considerations for implementation include ensuring reactor material compatibility, managing operational parameters such as pH and temperature, and scaling up from laboratory to industrial scale. The integration is expected to reduce requirements for external energy and improve overall system efficiency. However, challenges such as balancing power density with COD removal efficiency and managing increased system complexity need to be addressed.

In terms of scalability, attention must be given to material durability, particularly of electrodes and membranes, to withstand long-term operational stresses. Additionally, maintaining stable microbial communities over extended periods is crucial for sustained performance. Furthermore, the increased complexity of integrated systems may necessitate advanced monitoring and control strategies to ensure consistent operation at a larger scale. The development of submergible and stackable MFCs or electrode modules, as highlighted by Tan *et al.* (*32*), could also play a crucial role in improving scalability and operational flexibility. Moreover, integrating membrane-less systems or low-cost membranes could further reduce costs and improve the feasibility of large-scale deployment (*32*).

In terms of economic considerations, both MEC and MFC have similar capital costs due to their similar architecture in this experiment. Moreover, they are environmentally friendly and safe, as they do not require toxic chemicals to facilitate reactions. Additionally, these technologies are not labour-intensive and do not require large operational areas. However, it is important to note that the cycle time for both processes is around 3 to 4 days to produce sufficient bioenergy. This relatively long cycle time could affect the efficiency and scalability of these systems, potentially leading to increased operational costs and slower throughput. Efficient integration of these technologies into existing wastewater treatment infrastructure would benefit from addressing these cycle time challenges to optimise performance and cost-effectiveness.

In summary, integrating MEC and MFC technologies is a highly feasible concept for sustainable bioenergy production and wastewater treatment. The experimental results provide valuable insights into the operational parameters, efficiency and potential benefits of this integrated approach. Further research and optimisation efforts are crucial to improve process efficiency, reduce cycle times and address key factors for scalability and long-term stability. Investigating strategies for integrating MEC and MFC technologies into existing wastewater treatment infrastructure will be essential for their practical application. Additionally, future research should include additional parameters such as volatile fatty acids (VFAs), total nitrogen and other soluble organics, in addition to pH and COD, to offer a more comprehensive understanding of effect of substrate.

## CONCLUSION

This study concludes that dual-chamber reactors, separated by a proton exchange membrane, are effective in hydrogen production in microbial electrolysis cells (MEC) and generation of electricity in microbial fuel cells (MFC) for the treatment of palm oil mill effluent (POME) and production of bioenergy. The MECs successfully produced hydrogen gas with a maximum yield of 3.135 m^3^ H_2_ per kg of chemical oxygen demand (COD) removed and removed 48.7 % COD at an influent COD value of 150 mg/L and a voltage supply of 0.338 V. In addition, the MFCs produced electricity and reached a maximum power density of 2.10 mW/m^2^, a voltage of 0.20 V and a current density of 10.43 mA/m^2^ at a resistance of 5100 Ω. These results highlight that low influent COD value and low applied voltage improve hydrogen production by maintaining microbial activity and reducing inhibitory effects. Furthermore, the MFC operating with a 5100 Ω resistor were able to generate sufficient voltage (0.3 V) to power the MEC without external energy input, showing the potential for improving the sustainability and cost-effectiveness of the system. However, despite these promising outcomes, several challenges remain for scaling up. The optimum conditions observed at low COD value may not directly translate to industrial effluents, which typically have higher and more variable COD values that could affect system stability and performance. Long-term biofilm stability and microbial community control under fluctuating conditions remain critical concerns. Compared to previous research, this work introduces a novel integration of MFC and MEC systems using real POME as a substrate, offering a low-carbon pathway for waste-to-energy applications in the palm oil industry. The obtained insights provide a foundation for a scale-up, which will require addressing operational issues like mass transfer limitations, electrode fouling and energy balance. Future work should focus on the optimisation of system integration, biofilm robustness and microbial dynamics to improve resilience and energy recovery. Investigating additional parameters such as volatile fatty acids, nitrogen and other organic materials will extend the understanding of the effects of substrate. Efforts to integrate these technologies into existing treatment infrastructure will be vital for practical application.

## Appendix

**Table S1:** ANOVA for the model of hydrogen production rate (*Q*) and chemical oxygen demand (COD) removal efficiency (*η*_COD_)

**Source**	**Sum of squares**	**DF**	**Mean square**	**F-value**	**p-value**
***Q*(H_2_) (2FI model)**					
**Model**	1227.14	3	409.05	201.00	<0.0001
**A: COD as *γ*(O_2_)**	75.17	1	75.17	36.93	0.0017
**B: *V*_supplied_**	327.02	1	327.02	160.69	<0.0001
**AB**	45.90	1	45.90	22.55	0.0051
**Residual**	10.18	5	2.04		
**Lack-of-fit**	10.18	1	10.18		
**Pure error**	0.0000	4	0.0000		
**Total**	1237.32	8			
***η*_COD_ (Linear model)**					
**Model**	2803.80	2	1401.90	80.05	<0.0001
**A: COD as *γ*(O_2_)**	2046.02	1	2046.02	116.84	<0.0001
**B: *V*_supplied_**	366.26	1	366.26	20.92	0.0038
**Residual**	105.07	6	17.51		
**Lack-of-fit**	105.07	2	52.54		
**Pure error**	0.0000	4	0.0000		
**Total**	2908.87	8			

**Table S2:** Microbial electrolysis cell optimised value from Design-Expert software

**No.**	**Influent COD as *γ*(O_2_)/(mg/L)**	***V*_supplied_/V**	***Q*(H_2_ production)/(mL/day)**	***η*_COD_/%**	**Desirability**
**1**	150.001	0.338	34.800	48.680	0.966
**2**	150.001	0.304	34.532	49.142	0.963
**3**	150.000	0.274	34.290	49.557	0.961
**4**	150.001	0.229	33.931	50.176	0.957
**5**	150.000	0.222	33.875	50.272	0.956
**6**	150.005	0.193	33.646	50.666	0.953
**7**	150.002	0.189	33.611	50.725	0.953
**8**	150.001	0.596	36.855	45.146	0.942
**9**	150.001	0.681	37.535	43.975	0.934
**10**	150.004	0.863	38.984	41.482	0.916
**11**	150.003	0.895	39.240	41.042	0.912
